# A regional genomic surveillance program is implemented to monitor the occurrence and emergence of SARS-CoV-2 variants in Yubei District, China

**DOI:** 10.1186/s12985-023-02279-6

**Published:** 2024-01-08

**Authors:** Fangyuan Liu, Peng Deng, Jiuhong He, Xiaofeng Chen, Xinyu Jiang, Qi Yan, Jing Xu, Sihan Hu, Jin Yan

**Affiliations:** Chongqing Yubei Center for Disease Control and Prevention, Chongqing, China

**Keywords:** SARS-CoV-2, Epidemiology, Phylogenetics, Genomic surveillance, Bayesian analysis, Whole genome amplicon sequencing, YuBei

## Abstract

**Background:**

In December 2022, Chongqing experienced a significant surge in coronavirus disease 2019 (COVID-19) epidemic after adjusting control measures in China. Given the widespread immunization of the population with the BA.5 variant, it is crucial to actively monitor severe acute respiratory syndrome coronavirus 2 (SARS-CoV-2) variant evolution in Chongqing's Yubei district.

**Methods:**

In this retrospective study based on whole genome sequencing, we collected oropharyngeal and nasal swab of native COVID-19 cases from Yubei district between January to May 2023, along with imported cases from January 2022 to January 2023. Through second-generation sequencing, we generated a total of 578 genomes.

**Results:**

Phylogenetic analyses revealed these genomes belong to 47 SARS-CoV-2 Pango lineages. BA.5.2.48 was dominant from January to April 2023, rapidly replaced by XBB* variants from April to May 2023. Bayesian Skyline Plot reconstructions indicated a higher evolutionary rate (6.973 × 10^–4^ subs/site/year) for the XBB.1.5* lineage compared to others. The mean time to the most recent common ancestor (tMRCA) of BA.5.2.48* closely matched BA.2.75* (May 27, 2022). Using multinomial logistic regression, we estimated growth advantages, with XBB.1.9.1 showing the highest growth advantage (1.2, 95% HPI:1.1–1.2), followed by lineage FR.1 (1.1, 95% HPI:1.1–1.2).

**Conclusions:**

Our monitoring reveals the rapid replacement of the previously prevalent BA.5.2.48 variant by XBB and its sub-variants, underscoring the ineffectiveness of herd immunity and breakthrough BA.5 infections against XBB variants. Given the ongoing evolutionary pressure, sustaining a SARS-CoV-2 genomic surveillance program is imperative.

**Supplementary Information:**

The online version contains supplementary material available at 10.1186/s12985-023-02279-6.

## Background

Around December 2019, a respiratory virus began spreading among individuals in Wuhan, Hubei Province, China. This virus, known as the “2019 novel coronavirus” (2019-nCoV), became the source of a global pandemic [1]. As of early June 2023, there have been reports of nearly 520 million infections worldwide, with a death toll exceeding 6 million [2]. The World Health Organization (WHO) has been monitoring the genomic evolution of the severe acute respiratory syndrome coronavirus 2 (SARS-CoV-2) and updating the list of variants of concern (VOCs), variants of interest (VOIs) and variants under monitoring (VUMs) that are of particular interest [3]. These variants are anticipated or already known to have genetic alterations that can impact the viral characteristics related to transmissible [4–6], disease severity [7, 8], immune evasion [9–11], and resistance to diagnosis or treatment [12]. Since the emergence in 2019, SARS-CoV-2 has produced many variants after rapid evolution, including Alpha, Beta, Gamma, Delta, and Omicron [13]. Alpha variant has mutations that increase ACE2 binding and transmissibility [14, 15]. Beta and Gamma variants partially escape vaccine and infection-induced immunity [16–18]. Delta variant has key mutations that increase infectivity [19]. Omicron variant has numerous mutations that significantly evade vaccine-induced immunity [20, 21]. In March 2023, WHO updated the definitions for VOCs, VOIs and VUMs. BA.2.75  and XBB.1.9.1 were defined as VUMs and XBB.1.5 and XBB.1.16 were categorized as VOIs, tracing the origin and spreading patterns of these SARS-CoV-2 variants is critical for evaluating and guiding the measures to improve the impact of the virus on public health.

Towards the end of 2019, China reported the first complete genome sequence of SARS-CoV-2 to the WHO [22]. The Global Initiative on Sharing All Influenza Data (GISAID) advocates for the rapid sharing of data on all influenza viruses and coronaviruses causing COVID-19. This includes genetic sequences associated with human viruses, as well as relevant clinical and epidemiological data, aiding researchers in understanding the virus's evolution and transmission during epidemics and pandemics [23]. By May 2023, over 15 million SARS-CoV-2 genomes have been made available. The global genomic resources have enabled researchers to deepen their understanding of the pandemic, supporting close monitoring of the emergence of genomic diversity and the identification of functional characteristics of these new strains [24]. From a public health perspective, real-time whole-genome sequencing (WGS) of SARS-CoV-2 can track transmission patterns through genomic epidemiology and provide a deeper understanding of the virus's pathogenesis and virulence through comparative genomic analysis. It may also support the development of targeted vaccines and drugs [25].

The Omicron variant, with multiple lineages including BA.1, BA.2, and BA.3, emerged in November 2021. Subsequently, the BA.1 lineage rapidly disseminated globally, outcompeting other variants of concern and causing a surge in cases. By late April 2022, BA.2 had superseded BA.1 due to even higher transmissibility, although it did not appear to cause more severe disease compared to prior variants [26, 27]. In South Africa, BA.5 emerged in early 2022 and demonstrated better evasion of immunity from vaccination or prior infection than BA.1, thereby sparking a fifth wave of the coronavirus pandemic [28]. From late 2022 until now, Omicron subvariants have extensively diversified, with XBB subvariants circulating globally, posing a serious threat to current COVID-19 vaccines. China has also been overrun by XBB subvariants. Previously, the BF.7.14 and BA.5.2.48 lineages independently evolved in China, dominating from late 2022 to early 2023. However, they were swiftly displaced by XBB as these variants did not acquire additional immune-escape mutations of concern [29]. As SARS-CoV-2 variants spreads amid growing population immunity, its tendency for antibody escape has become a key determinant of variant fitness [30]. This enables Omicron to readily infect those vaccinated or previously infected, and undergo adaptive evolution under selection pressures imposed by antibodies and therapeutics [31]. Therefore, in the background of herd immunity, Omicron exhibits better immune evasion, it is necessary to dynamically detect the prevalence of SARS-CoV-2 variants. Chongqing, China, has established an early warning system for epidemic surveillance, closely monitoring the spread of the virus. Stringent control measures have reduced population mobility and effectively cut off transmission pathways, allowing for early control and containment of the outbreak. Yubei, located at an airport transportation hub with frequent population movement, serves as a microcosm of Chongqing's situation regarding the prevalence of SARS-CoV-2 variants. However, the dynamic transmission and genetic evolution characteristics of SARS-CoV-2 variants, especially the dominant strains BA.5.2.48 and XBB, in the Yubei district of Chongqing remain unclear. In December 2022, China adjusted its epidemic prevention policies, starting from 2023, Chongqing Yubei Center for Disease Control and Prevention (CDC) as a member of Chinese CDC SARS-CoV-2 surveillance network, we collected and sequenced SARS-CoV-2 samples on a weekly basis from various sentinel sites. The quantity and frequency of sample collection will impact the effectiveness of the surveillance program. According to a regional SARS-CoV-2 genomic surveillance project conducted by a metropolitan hospital in St. Louis, Missouri, USA, a sampling rate of approximately 5 samples per 1,000,000 people per week is sufficient to detect the prevalence of both known and novel VOCs in the community [32].

In this research, we analyzed 578 whole genomes of SARS-CoV-2 collected from January 2022 to January 2023 from the local region of Yubei district of Chongqing and imported from overseas, aiming to track the dynamic changes in the transmission and monitor the genetic evolution characteristics of SARS-CoV-2 in Yubei district of Chongqing. We seek to promptly identify emerging variants and mutations of SARS-CoV-2, particularly observing the prevalence of these variants amidst the establishment of population immunity barriers, which may provide valuable data for the monitoring and control measures of variants in Yubei district of Chongqing.

## Materials and methods

### Sample and data sources

The nasal or pharyngeal swab specimens from COVID-19 infected individuals for this study were collected from January 2022 to May 2023, spanning customs checkpoints, quarantine hotels, third-party testing agencies, sentinel hospitals, and three adjacent regional centers for disease control and prevention. All collected samples were sent to Yubei district of Chongqing CDC for verification and further sequencing. A total of 603 positive samples were enrolled from confirmed SARS-CoV-2 cases for genomic sequencing.

Samples were required to be submitted weekly by each unit. Patient metadata, such as age, gender, sample source (domestic province or foreign country), and other relevant information, were collected for all samples. Upon receipt, the laboratory was storing the samples at either  − 70 °C or 4 °C, depending on the experimental timeline. Before sequencing, the samples need to undergo real-time reverse transcription PCR (RT-PCR) testing. The total viral RNA was extracted from 200 μL of sample using the nucleic acid extraction kit (Zybio, China) and eluted using 50 μL RNase-free water. The bead clean-up was performed according to protocols provided by the manufacturer (Zybio, China). The 10 μL eluted water of each sample was conducted to RT-PCR using the Da An Gene Nucleic Acid Extraction Kit according to the manufacturer’s instructions (Daan, China). The remaining extraction steps were performed using the Thermo Scientific Flex System. Samples with cycle threshold (Ct) values < 32 were selected for further experimentation to improve the sequencing success rate. Sample collection, transportation, nucleic acid extraction, and RT-PCR procedures adhered to the guidelines outlined in the New Coronavirus Pneumonia Prevention and Control Program by the National Health Commission of China.

### Genomic sequencing and assembly

The purified total RNA was rapidly and consistently converted into complementary DNA (cDNA) using a commercially available reagent kit, namely the SARS-CoV-2 whole-genome multiplex PCR kits (MicroFuture, Beijing, China; Baiyi Technology Co., Ltd., China). Subsequently, the SARS-CoV-2 genome was amplified in its entirety through segmented amplification using highly specific primers designed in a stacked-tile fashion and high-fidelity enzymes,which produce 1200 bp amplicons. The resulting products were prepared into libraries following the instructions provided in the VAHTS® Universal Plus DNA Library Prep Kit for Illumina (Vazyme Biotech Co., Ltd., China). After purification using AMPure XP (Beckman Coulter, USA), the libraries were quantified using the Qubit 3.0 fluorometer (Life Technologies, Austin, TX, USA), and samples with concentrations below or equal to 4 ng/µL were discarded [33]. The normalized libraries were then subjected to sequencing on the Illumina NextSeq2000 platform. The sequences preprocess including remove short, low-quality, and chimeric reads of raw reads, consensus genome assembly and variant calling of the genomes were performed using the Iterative Refinement Meta-assembler (IRMA) version1.0.3 [34] with the Wuhan-Hu-1 (Accession Number: NC_045512.2) as the reference genome. The resulting genomes were subjected to quality control and classified into clades using Nextclade version2.14.1 (https://clades.NextStrain.org/) [35], retaining only those with scores ranging from 0 to 99 for subsequent analysis. Samples that did not meet the quality control criteria underwent a comprehensive examination by plugins in IRMA to analyze heterozygous and private site profiles.

Prior to phylogenetic analysis of all genomes, reads were mapped to the reference genomes (Accession Number: NC_045512.2) to obtain BAM files by the BWA version 0.7.17software [36], then we performed a manual contamination investigation and recombination analysis of the BAM files using IGV version2.15.1 software [37], this enables us to inspect coverage and variation visually across the genome, during this process, our lab have reported the first case of co-infection with Omicron subvariants BA.5.2.48 and BF.7.14 in Chongqing [38].

### Phylogenetic analysis

NextStrain version6.2.1 was used for phylogeny analysis and the resulting phylogeny tree was visualized using Auspice (https://auspice.us/) [39]. We used SARS-CoV-2 workflow in the NextStrain to create phylogenetic trees of SARS-CoV-2 genomes [39]. Augur as an internal component of the Nextstrain's phylodynamic pipeline, was used to perform phylogenetic analysis [40]. Augur consisted of Python script that processed prepared sequences and metadata to produce an annotated phylogeny for visualization in Auspice. To reconstruct the phylogenetic tree between our sequenced genomes with those of other countries and regions around the world, we downloaded sequences with high-quality, coverage and complete genomics from GISAID, and then used Uvaia version2.0.1 [41] software to search for the top four closest neighboring sequences available in GISAID to each sequenced sample, a total of 202 genome from global were obtained.

Multiple sequence alignment was conducted by MAFFT version7.52 [42]. IQ-TREE version2.2.2.3 was used to reconstruct phylogenetic trees with 1000 ultrafast bootstrap replicates [43]. Then, the best‐fitting nucleotide substitution model for all sequenced genomes was selected by using the ModelFinder software based on the Bayesian Information Criterion (BIC). The Bayesian Evolutionary Analysis by Sampling Trees (BEAST) version1.10.4 software [44], was used to perform the Time-scaled Bayesian phylogenetic analyses. In our model, specified a Markov chain Monte Carlo (MCMC) length of 200 million generations, with sampling every 2,000 steps under uncorrelated relaxed clock model and strict clock model. For each run of 200 million of MCMC, path sampling (PS) and stepping stone (SS) methods were applied to estimate marginal likelihood and by computing the log marginal likelihood results to select the best‐fitting clock model among six clock-tree model combinations (constant size, exponential growth, and Bayesian skyline under uncorrelated relaxed clock model and strict clock model, respectively) to select the best model [44].

To infer the substitution rates and timescale of SARS-CoV-2 Omicron variants, Bayesian skyline inference analyses with the uncorrelated log-normal relaxed clock model were performed on the datasets of BA.5.2.48 and its descendant’s (i.e., BA.5.2.48*) (n = 203), BA.2.75 and its descendant’s (i.e., BA.2.75*) (n = 78), XBB.1.9.1 and its descendant’s (i.e., XBB.1.9.1*) (n = 186), XBB.1.5 and its descendant’s (i.e., XBB.1.5*) (n = 31), and XBB.1.16 and its descendant’s (i.e., XBB.1.16*) (n = 28) sub-lineages. We employed Treetime version 0.9.6 to conduct root-to-tip regression analysis, which provides an estimate of the evolutionary rates (substitutions per site per year), and the mean time to the most recent common ancestor (tMRCA) [45]. TN + F + I + R3, TN + F + I, TIM + F + I, TN + F, and HKY + F models were found to be the best nucleotide substitution models for BA.5.2.48*, BA.2.75*, XBB.1.9.1*, XBB.1.5*, and XBB.1.16* sub-lineages, respectively. The time-scaled maximum clade credibility (MCC) tree was annotated by the TreeAnnotator version1.10.4 software after a 10% burn-in [46]. Tracer version 1.7.2 software was used to diagnose MCMC inspected for convergence by imported the BEAST log files and the ESS values > 200 indicated the convergence is reliable [47]. The phylogenetic trees were edited and visualized using FigTree version1.4.0 (http://tree.bio.ed.ac.uk/software/figtree/) and the iTOL tool (https://itol.embl.de/). Genomes included in this research were filtered for high quality and coverage.

We also apply a Multinomial Logistic Regression (MLR) model to estimate frequencies and growth advantages of SARS-CoV-2 variants. The results were visualized in: https://Nextstrain.github.io/forecasts-viz/.

## Results

**Fig. 1:**
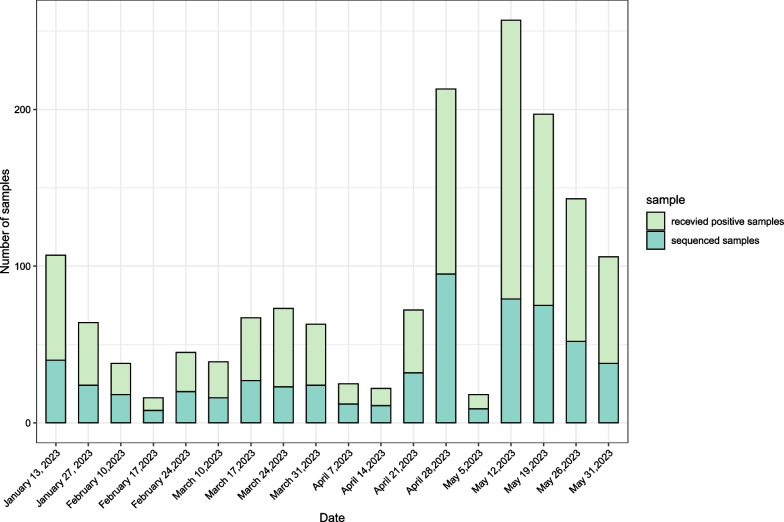
Proportion of samples collected and successfully sequenced

**Fig. 2:**
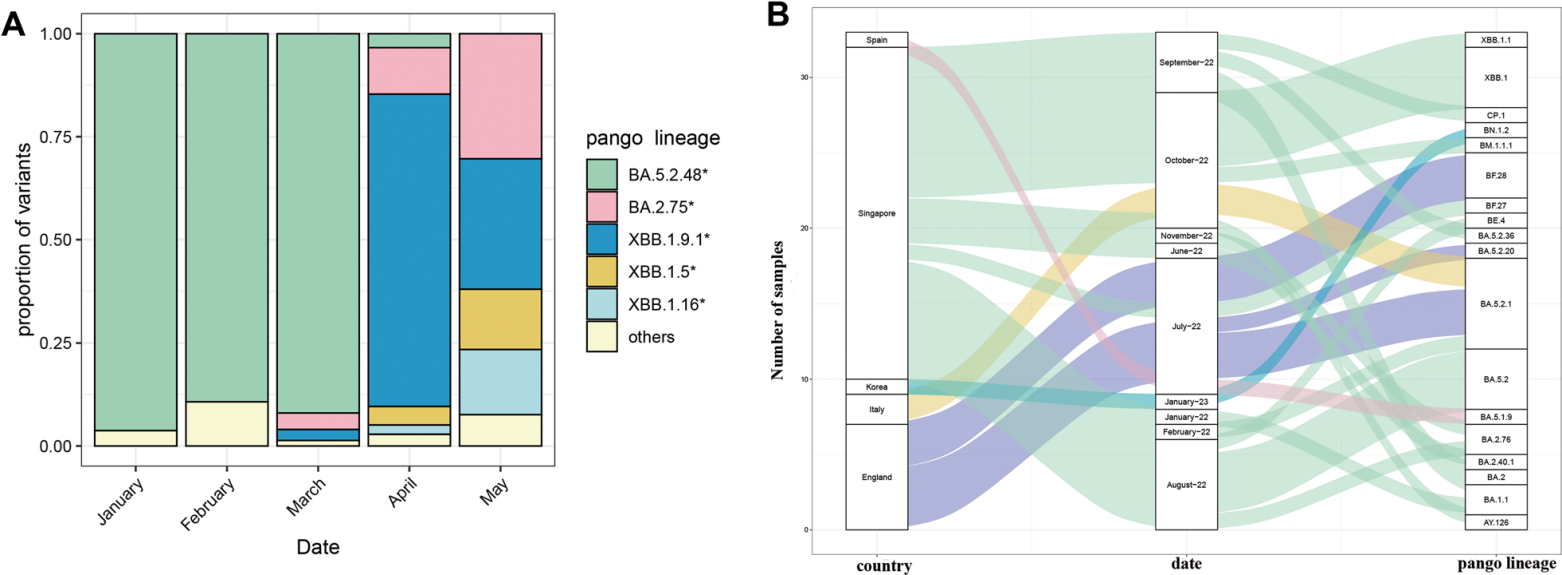
**A** The boxplot of distribution of different SARS-COV-2 lineages in Chongqing Yubei from January to May 2023. **B** The sankey diagram of imported distribution of the origin, sampling date and types of variants from January 2022 to January 2023

Between January 1, 2022, and May 30, 2023, a total of 962 SARS-CoV-2 positive samples were received, and 603 samples were successfully sequenced. After whole-genome sequencing and quality controls, 578 genomes from Chongqing Yubei district and surrounding area (n = 545) from Jan 1 to May 30, 2023 and overseas imported cases (n = 33) from Jan 1, 2022 to Jan 30, 2023 were included for further analysis (Fig. 1). Most local samples were collected in the Yubei district of Chongqing, and all imported samples came from 5 countries and regions (Fig. 2B). The details of the samples and the sequences obtained information are shown in Additional file 3: Table 1. The average age of all sequenced samples was 45.7 years. The male to female sex ratio was 1.1.

**Fig. 3:**
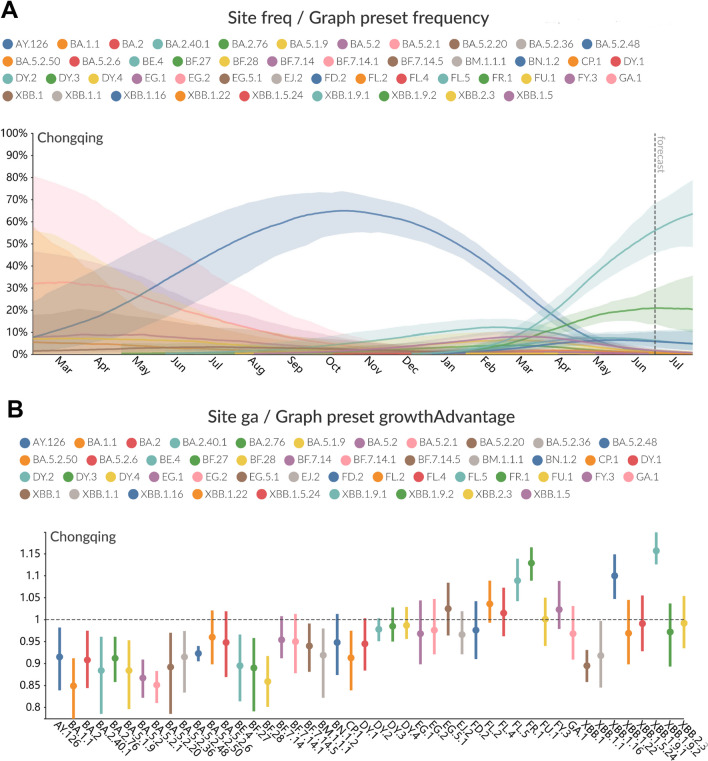
**A** Lineage frequencies over time. Each line represents the estimated frequency of a particular pango lineage through time. In multinational logistic regression, each curve represents the change in the proportion of a given lineage over time. The shaded part represents the 95% confidence intervals of estimated. Only the estimated frequencies of variant were the top five are shown. The deadline of forecast model is July 14, 2023. **B** Lineage growth advantage. the estimated growth advantage for given pango lineages relative to lineage XBB.1.5. Vertical bars show the 95% HPD

In this research, a total of 578 SARS-CoV-2 genomes was carried out phylogenetic analysis using NextStrain. The phylogenetic trees revealed that the genomes from different parts of Chongqing could be classified under 4 clades (22B, 21L, 21 K, and 21 J) (Fig. 2A) and 47 pango lineages, including 18 pango lineages from imported samples (Fig. 2B). All clades mainly clustered to two distinct clades, 22B, 21L and other clades. In the local samples, the proportion of lineages BA.5.2.48* (n = 119, 21.83% [95% CI 17.3–23.9]) were found to be dominated during January to March, 2023. In March, BA.2.75* identified were began to increase and continued to increase until May, which reach to 71 (13.03% [95% CI 9.6–15.0]). However, in April, the proportion of BA.5.2.48* was observed decreased, XBB* became the dominant variant during April to May, especially XBB.1.9.1* (n = 143, 26.24% [95% CI 21.2–28.3]), followed by XBB.1.5* (n = 30, 5.50% [95% CI 3.4–7.0]) and XBB.1.16* (n = 26, 4.77% [95% CI 2.8–6.2]) (Fig. 3A). Among the variants identified after April, XBB.1.9.1 and its sublineages demonstrate the highest prevalence. Between January 28, 2022 to January 31, 2023, 18 different lineages were identified in the imported samples, and dominated by BA.5.2.1, BA.5.2, and XBB.1 variants, most samples imported from *Singapore* (Fig. 3B).

**Fig. 4:**
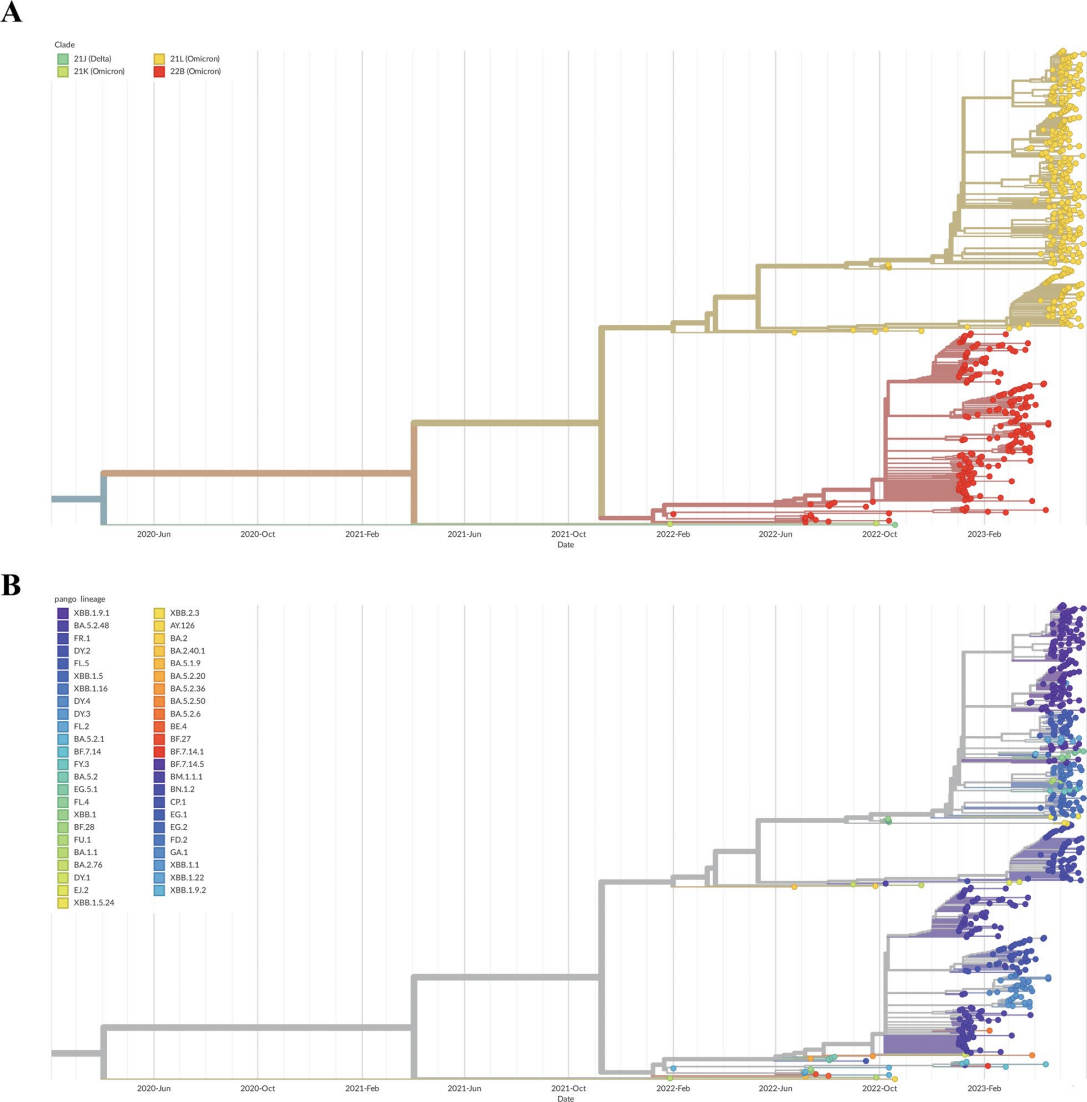
Phylogenetic tree in the time-scaled of 578 SARS-CoV-2 genomes sampled between January 2022 and May 2023 classification by clade (**A**) and classification by pango lineage (**B**). SARS‐CoV‐2, severe acute respiratory syndrome coronavirus‐2

In addition, the forecasts-ncov workflows in a multinomial logistic regression was used to estimate the prevalence of SARS-CoV-2 lineages. MLR model forecast indicated a decline trend in the frequency of BA.5.2.48 between January 2023 and May 2023, which was also consistent with our actual monitoring results. And the estimated frequency of top5 variants were XBB.1.9.1 (63.4%), FR.1 (20.5%), XBB.1.16 (5.0%), FL.5 (4.9%), and XBB.1.5 (0.9%) at July 14,2023 (Fig. 4A). We have evaluated the estimated growth advantage for given pango lineages relative to lineage XBB.1.5. The results indicated that there were 8 variants (XBB.1.9.1, FR.1, XBB.1.16, FL.5, FL.2, FL.4, EG.5.1, FY.3) had higher growth advantage when compared to lineage XBB.1.5, and lineage XBB.1.9.1 had the highest growth advantage (1.2, 95% HPI:1.1–1.2), followed by the lineage FR.1 (1.1, 95% HPI:1.1–1.2) (Fig. 4B).

**Fig. 5:**
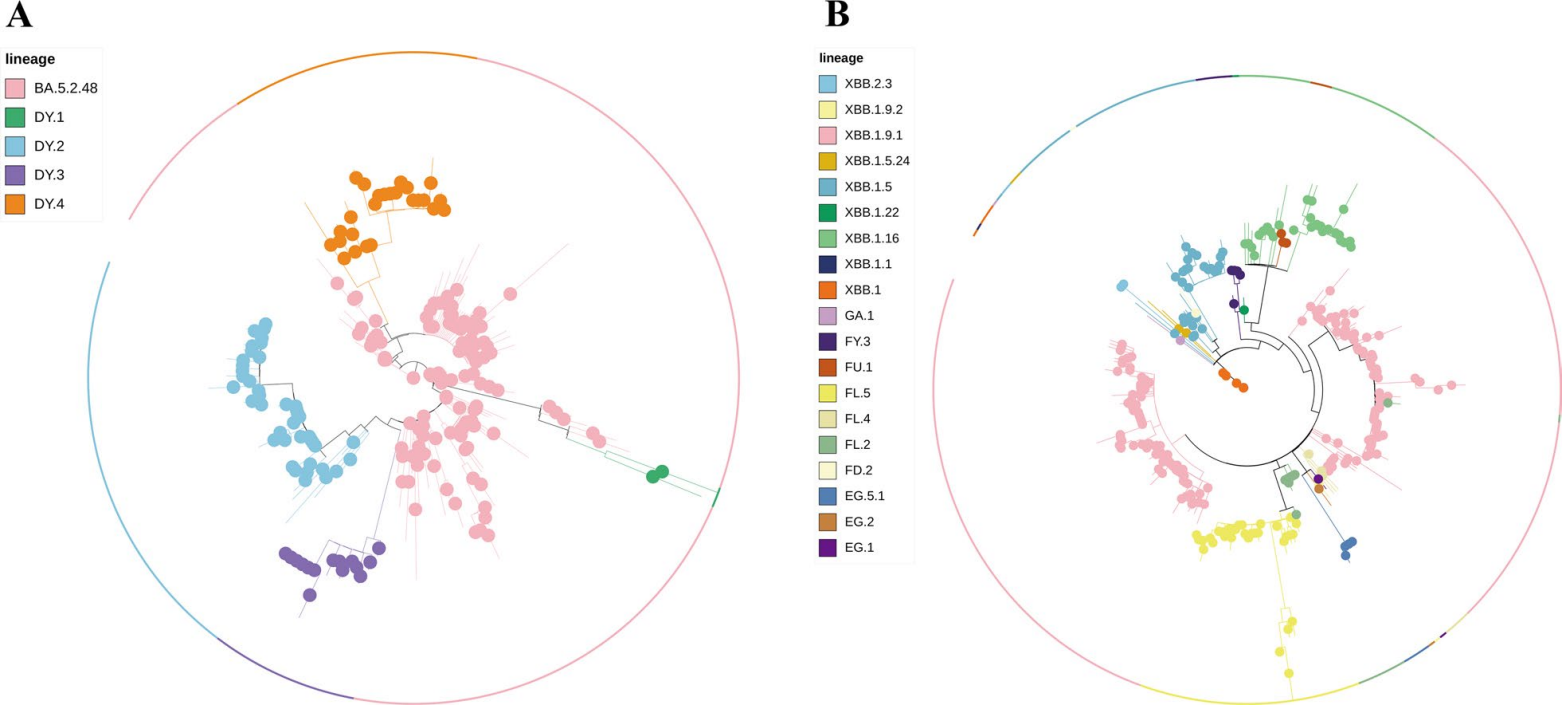
**A** Phylogenetic tree based on the full-length genome sequences of the Omicron Variant BA.5.2.48 and its descendants. **B** Phylogenetic tree based on the full-length genome sequences of the Omicron Variant XBB and its descendants. The sub-lineages are represented in the upper left corner

Phylogenetic analyses indicated that the BA.5.2.48 and its descendant’s variant formed five distinct lineages, with lineage BA.5.2.48, DY1, DY2, DY3 and DY4 (Fig. 5A) and the XBB with its descendant’s variant formed 19 distinct lineages (Fig. 5B). NextStrain analysis with genomes download from GISAID (n = 202) inference of the most likely transmission events revealed that the dominant lineage BA.5.2.48 was clustered with genomes from Zhejiang, Fujian, Taiwan and Guangxi, etc., suggesting the spread of infections between these divisions. Lineage DY.2 was divided into two clusters, one of which contained only four local samples, clustered with the genomes mainly from Hainan and Shandong, etc., while most of sequences were assigned to the second cluster, they were more closely related to genomes from Jiangsu and Zhejiang, etc., And lineage DY.3 clustered with the Sichuan genome, suggesting possible transmission events. In general, phylogenetic tree suggested that some cases emergence of lineage BA.5.2.48* may be associated with the movement of other provinces and have caused local transmission event. Cluster analysis demonstrated that the BA.5.2.48 variant and its sub-lineages, predominantly originated from regions outside the Yubei district and its adjacent areas within China. The significant degree of clustering signifies ongoing local transmission and spillover (Additional file 2: Fig. S2). Furthermore, the genomes of lineage BF.7.14 formed a separate cluster, and mainly clustered with the genomes of Zhejiang and Sichuan, FR.1 was clustered with genomes from Sichuan, Yunnan, Shanghai, and Henan, which indicated that the omicron subvariants may have closely association among different provinces and cities. Meanwhile, we observed that all FR.1 sequences were highly similar and were branching from a sample belong to BN.1.2, our finding indicated that potential linkage between this case belong to BN.1.2 and local cases of lineage FR.1 in Yubei. In October 2022, XBB was a relatively dominant variant internationally at that time, in our study, the sequences of lineage XBB.1 and XBB.1.1 were foreign imported cases we identified that they were grouped into a single cluster, and didn’t cause widespread transmission, associated with China implemented strict border control measures. The XBB.1.9.1 was shared similarities to genomes derived from the India and Germany. The FL.2 FL.4, and FL.5 were mainly clustered with the Hubei genomes. All in all, the sequences from Chongqing Yubei district were clustered with some sequences collected in other countries or provinces, suggesting that they had the same source (Additional file 2: Fig. S2).

In this sutdy, phylogeny analysis based on the Bayesian skyline under the log-normal uncorrelated relaxed clock model were carried out on the aligned sequences to identify the effective population size for BA.5.2.48*, BA.2.75*, XBB.1.9.1*, XBB.1.5*, and XBB.1.16* variants, which indicating different change trend in genetic diversity within these five lineages (Fig. 5). The Bayesian skyline plot (BSP) showed that the effective population size of BA.5.2.48* increased sharply around December 10, 2022, and achieved the highest at December 26, 2022, then gradually declined until reaching a plateau around February 9, 2023, which coincide with the outbreak of the Chongqing epidemic. The viral population of BA.2.75* showed no significant fluctuation between September 27, 2022 and April 11, 2023, while exhibited exponential growth from April 14, 2023, and reaching the peak at about April 26, 2023. The effective population size of XBB.1.9.1* experienced a rising trend from March 15, 2023 to May 25, 2023, and a sudden expansion occurred around April 17, 2023, then it reached a stable level around April 30, 2023, which was higher than the effective population size when the other three variants reached a stable level. BSP indicated that the effective population size of the lineages XBB.1.5* and XBB.1.16* exhibited a relatively stable trend, however, the effective population size of the lineages XBB.1.16* was higher than XBB.1.5*.

In our study, Treetime was employed to perform root-to-tip regression analysis on the ML trees to assess the temporal molecular evolutionary signals (Additional file 1: Fig. S1). Root to tip regression analysis demonstrate that the evolutionary rate was 4.492 × 10^–4^ subs/site/year (95% HPD: 2.852 × 10^–4^ − 6.132 × 10^–4^), 6.710 × 10^–4^ subs/site/year (95% HPD: 4.34 × 10^–4^ − 9.08 × 10^–4^), 5.609 × 10^–4^ subs/site/year (95% HPD:2.739 × 10^–4^ − 8.479 × 10^–4^), 6.973 × 10^–4^ subs/site/year (95% HPD: 0.163 × 10^–4^ − 1.3783 × 10^–3^), and 5.757 × 10^–4^ subs/site/year (95% HPD: − 2.893 × 10^–4^ − 1.4407 × 10^–3^) of BA.5.2.48*, BA.2.75*, XBB.1.9.1*, XBB.1.5*, and XBB.1.16* sub-lineages, respectively. And the tMRCA of BA.5.2.48*, BA.2.75*, XBB.1.9.1*, XBB.1.5*, and XBB.1.16* sub-lineages was May 27,2022 (95% HPD: February 18,2022 − September 3,2022), May 27,2022 (95% HPD: February 22,2022 − August 30,2022), September 20,2022 (95% HPD: July 10,2022 − January 31,2023), November 26,2022 (95% HPD: July 10,2022 − April 14,2023) and February 7,2023 (95% HPD: August 26,2022 − July 21,2023), respectively. System dynamic reconstruction revealed that the common ancestor of the BA.5.2.48 variant emerged in September 2022, roughly four months prior to the earliest collected sample from September 2022. Bayesian skyline analysis suggested a comparatively limited genetic variability within the BA.5.2.48* variant, reaching its peak population size in December 26, 2022 and subsequently undergoing a swift decline. This finding was consistent with the predictions of the MLR model (Fig. 4). The estimated evolutionary rate derived from molecular clock calibration was the most minimal when compared to the major lineages under analysis.

## Discussion

This retrospective study analyzed the genomic data of the SARS-CoV-2 collected from January 2022 to May 2023 to investigate the introduction, transmission, and evolution of the SARS-CoV-2 in the Yubei district. Prior to December 2022, the implementation of effective domestic zero-COVID measures in Chongqing resulted in a majority of positive COVID-19 cases being associated with imported cases, a correlation strongly linked to the volume of international flights. Throughout this period, Chongqing witnessed a substantial decrease in flight volume, specifically originating from countries including Singapore, the United Kingdom, Italy, and South Korea. The imported SARS-CoV-2 variants from these countries were consistent with the international epidemic variants. A large number of studies have confirmed the contribution of air travel to the transmission of SARS-CoV-2. As Kanteh, Abdoulie et al. research described that the relaxation of restrictions on the air travel has led to an increase in the SARS-CoV-2 lineage diversity [33]. An early analysis of COVID-19 propagation also showed the significance of air travel in the propagation of the virus [48]. Yubei District, as the largest airport hub center and the largest social population district in Chongqing, may become the main source of the introduction and spread of new SARS-CoV-2 lineages and variants due to the large population flow brought about by air travel [49]. Singapore has a well-developed have international transportation network, which can be used to explain the diverse lineage composition of virus strains in imported sequences were in Singapore in our study [50], such as XBB.1, BA.1.1, CP.1, BM.1.1.1, XBB.1.1, and AY.126 and so on were imported from Singapore. We speculated that air travel provided a way to facilitate the transmission of the virus and the spread across regions in Yubei District of Chongqing [51]. Nevertheless, stringent isolation measures for incoming travelers prevented any instances of sustained local transmission resulting from imported cases.

According to our dynamic monitoring results, the prevalence of SARS-CoV-2 variants changed with time in Yubei District of Chongqing. Following the modification of China's epidemic prevention measures in December 2022, there was an increase in the frequency of domestic and international travel, resulting in a significant surge in COVID-19 infections in Chongqing. Previous research has reported in China, BA.5.2.48 was found to be most prevalent lineages from December, 2022 to January, 2023 [29, 52]. In our study, subsequent to mid-April, there was a decline in the prevalence of the BA.5.2 variant and its sub-lineages within the population. This could be attributed to the absence of novel mutations enabling immune evasion in the BA.5.2.48 variant and BF.7 variant under population immunity [29]. Besides, Time-scaled Bayesian phylogenetic analyses result indicates that the tMRCA of BA.5.2.48* was about September 2022, the BA.5.2 variant was first detected in February 2022, approximately six months prior to its widespread circulation in Chongqing. Sun, Yamin et al. also speculated that the variant circulation of BA.5.2.48 in China may have been imported in Beijing at the beginning of September 2022 [29], which agree with the assumption of the BA.5.2.48* introduction time in our study.

**Fig. 6:**
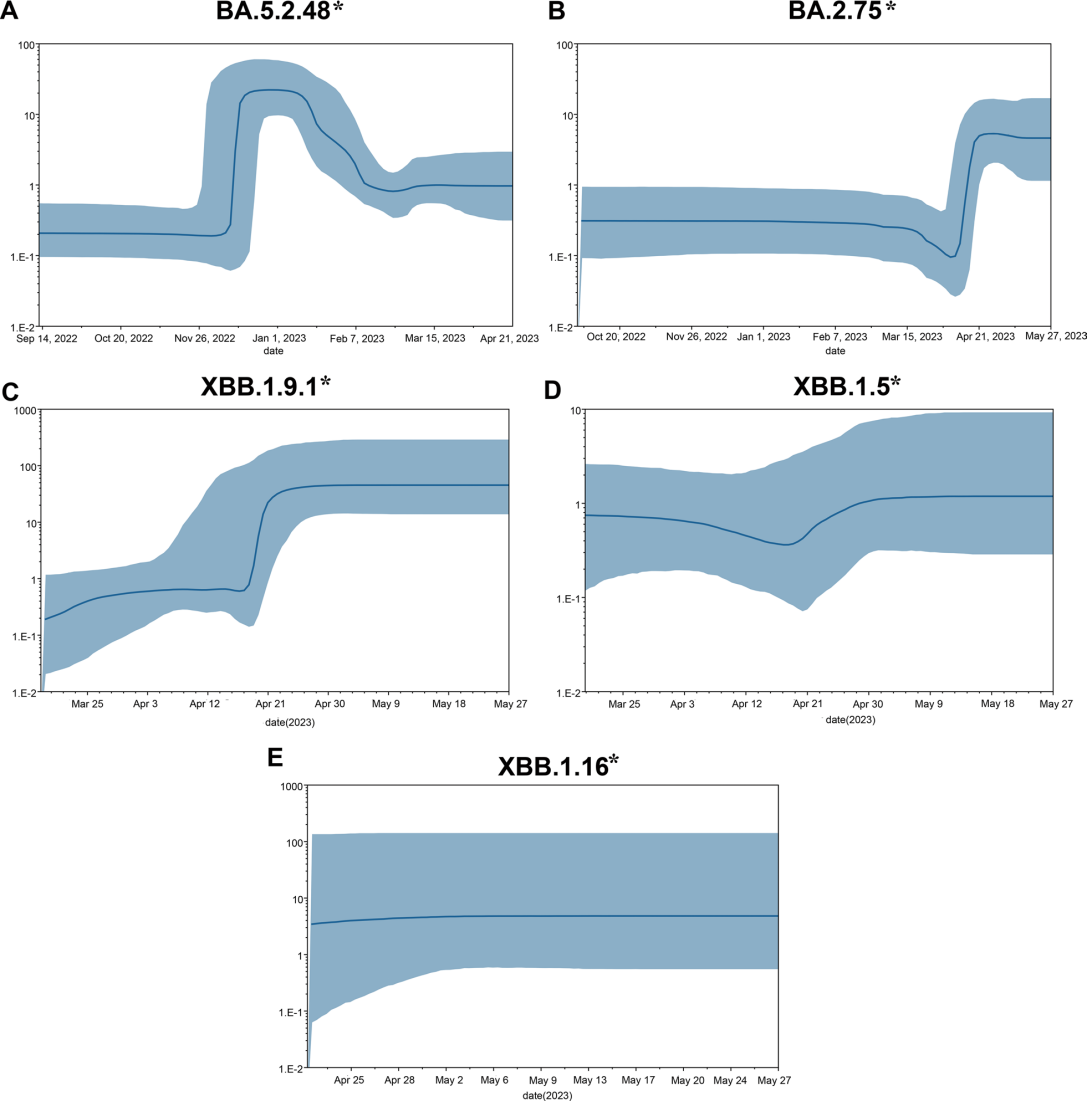
Bayesian skyline plot of SARS-CoV-2 variant BA.5.2.48 (**A**), BA.2.75 (**B**), XBB.1.9.1 (**C**), XBB.1.5 (**D**), and XBB.1.16 (**E**). The viral effective population size (y-axis) is shown as a function of days (x-axis). The thick solid line represents the median estimate and the solid area represents the 95% high posterior density (HPD) region

In 2023, within the Yubei district and its surrounding areas, the detection and complete replacement of the BA.5.2 variant by XBB lineages occurred in less than two months. Figure S2 demonstrated that the XBB lineages in the Yubei district and its neighboring regions are predominantly introduced from international sources. This outcome is an inevitable result of the restoration of international travel. It is noteworthy that this took place merely 3–4 months subsequent to the implementation of domestic immunity barriers, giving rise to the second surge of infections in Chongqing, consistent with the nationwide trend. XBB lineages represent recombined strains originating from two BA.2 lineages (BJ.1 and BM.1.1.1), leading to a substantial reduction in the serum's overall neutralization response as the antigenic disparity between the stimulating antigen and the wild-type antigen intensifies (XBB > BA.5) [53]. Additionally, the persistent emergence of XBB.1.5 [54, 55] and XBB.1.16 [56, 57], characterized by significant immune evasion, further exacerbates the second surge of infections in the Yubei district. Among the variants identified after April, XBB.1.9.1 and its sublineages demonstrate the highest prevalence. In contrast to XBB.1.5, XBB.1.9.1 exhibits no disparities in the S protein but does harbor a gain-of-function mutation (I5T) in ORF9b [58], potentially linked to the ORF-9b's inhibition of innate immunity through its interaction with mitochondria and the MAVS/TRAF3/TRAF6 signalosome [59]. One notable observation is that the mutation is also present in XBB.1.16, which may contribute to the preferential expansion of XBB.1.9.1 and XBB.1.16 within the circulating lineages of XBB lineages (Fig. 6). After the disappearance of BA.5.2, FR.1 emerges as the sole non-XBB lineage among the circulating variants, and excited relatively high estimated frequency and growth advantage (Fig. 4). FR.1 is derived from the BA.2.75 lineage, and studies have shown that N-terminal domain (NTD) mutations, K147E and W152R, in FR.1 result in resistance to neutralization by convalescent sera [59, 60]. FR.1 demonstrates enhanced binding affinity to the ACE2 receptor [14, 60], contributing to its competitive advantage. The evolutionary rates of multiple major XBB variants and the FR.1 variant were estimated through molecular clock calibration. Among them, XBB.1.5 exhibits the highest rate at 6.973 × 10^–4^ subs/site/year (95% HPD: 0.163 × 10^–4^ − 1.3783 × 10^–3^), which is still one order of magnitude slower than the Wuhan-Hu-1 variant [61]. XBB.1.5 variant has acquired a number of mutations derived from previous variants that give it a competitive advantage in both its binding to ACE2 receptors and its ability to escape antibodies [62]. Bayesian skyline reconstruction reveals a flattened trajectory for all XBB variants and FR.1 following a transient expansion (Fig. 6). Considering their prolonged circulation prior to detection, this pattern deviates from the characteristics of highly expanding variants [63]. Notably, the EG.5.1 variant, detected in May, exhibits a notable relative growth advantage despite its low frequency (Fig. 4). While EG.5.1 shares similarity with XBB.1.5 in the S protein, it carries an additional concerning mutation, Spike F456L, predicted as another beneficial immune escape mutation in XBB* based on deep mutational scanning experiments [64].

The present study possesses certain limitations. In 2023, the unavailability of samples from imported cases hindered our ability to perform a comparative analysis between local and imported cases' genomes. The clinical information of laboratory-confirmed COVID-19 cases is unavailable. Additionally, we did not meticulously quantify the correlation between the quantity of genomes and the number of cases, potentially introducing sampling bias [65]. The Bayesian system analysis relied on extensive and intensive sampling [66], which could lead to a certain degree of inaccuracy in our findings. This aspect further emphasizes the necessity of prolonged and scientifically rigorous monitoring of sampling to acquire more precise outcomes. While no new variations were observed in the RBD region during previous surveillance, the evolving pressures of the SARS-CoV-2 in the second wave of infections, occurring within a novel immune context (BA.5 → XBB*), may potentially result in novel mutations. Hence, the implementation of a dynamic and continuous monitoring program for the genomic sequence of the SARS-CoV-2 is of paramount importance.

## Conclusions

In conclusion, WGS analysis reveals that SARS-CoV-2 variants were transmitted in Yubei and its adjacent areas in Chongqing from January 2022 to May 2023, and the dominant strain changed from BA.5.2.48* to XBB* around April 2023. Our results provide phylogenetic information on the SARS-CoV-2 variant circulating in Yubei, Chongqing and its adjacent areas, revealed the ineffectiveness of herd immunity and breakthrough BA.5 infections against XBB variants.

### Supplementary Information

**Additional file 1**. **Fig. S1**. Root-to-tip regression analyses on the maximum likelihood (ML) trees generated for original dateset comprising BA.5.2.48 (**A**), BA.2.75 (**B**), XBB.1.9.1 (**C**), XBB.1.5 (**D**), and XBB.1.16 (**E**) lineages.

**Additional file 2**. **Fig. S2**. Phylogenetic tree in the time-scaled of 578 SARS-CoV-2 genomes sampled sequenced and 202 SARS-CoV-2 genomes sampled download from GISAID between January 2022 and May 2023 classification by divisions. GISAID, Global Initiative on Sharing All Influenza Data.

**Additional file 3**. **Table S1**. The sample information of all sequenced.

## Data Availability

The raw sequencing reads from this study have been submitted to the GenBank with the project Accession SUB13486524 and in the GISAID database at https://www.gisaid.org/.

## Abbreviations

COVID-19: Coronavirus disease 2019
GISAID: Global initiative on sharing all influenza data
SARS-CoV-2: Severe acute respiratory syndrome coronavirus-2
VOI: Variants of interest
VOC: Variants of concern
VUMs: Variants under monitoring
WHO: World Health Organization
tMRCA: The mean time to the most recent common ancestor
MLR: Multinomial Logistic Regression
