# Supplementary figures and images for: A regional genomic surveillance program is implemented to monitor the occurrence and emergence of SARS-CoV-2 variants in Yubei District, China
Source: Virol J. 2024 Jan 8;21:13. doi: 10.1186/s12985-023-02279-6 (PMC10775548; doi:10.1186/s12985-023-02279-6)

**A****BA.5.2.48\***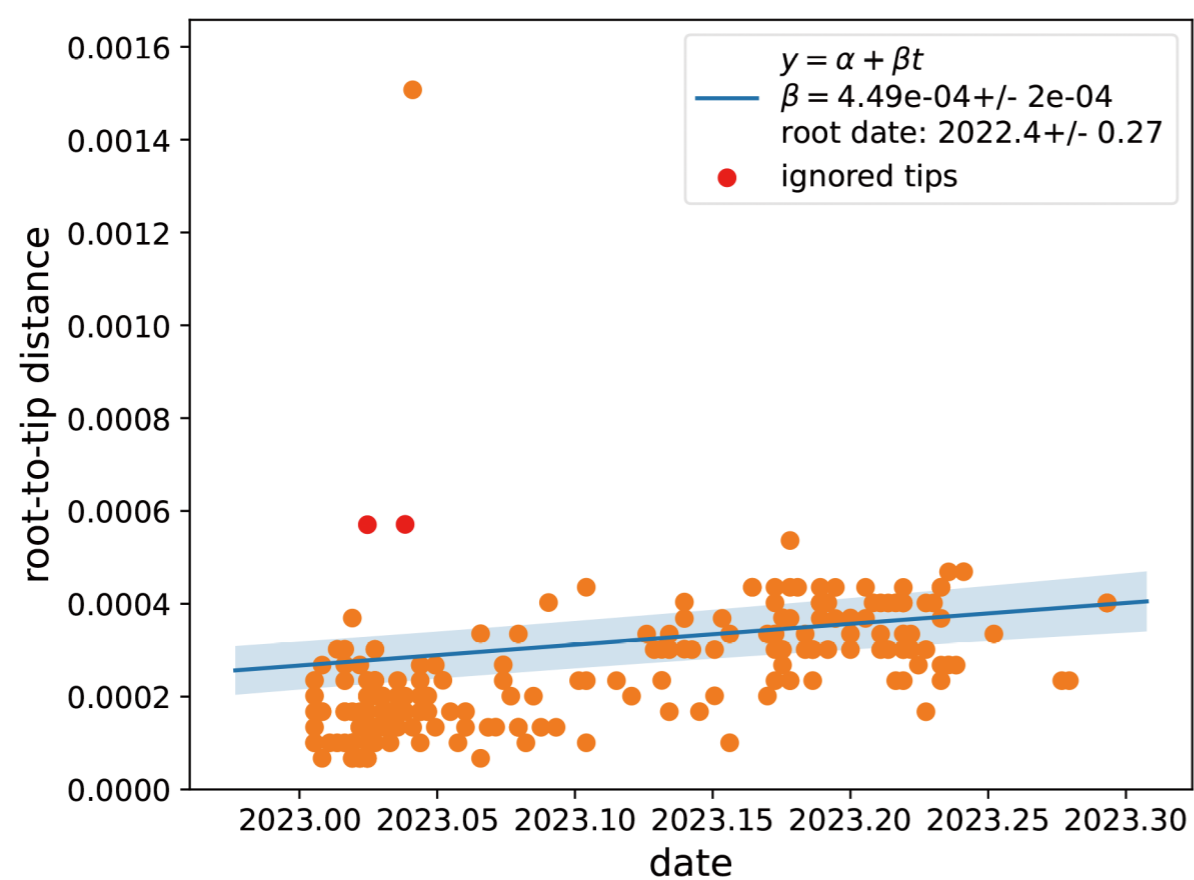**B****BA.2.75\***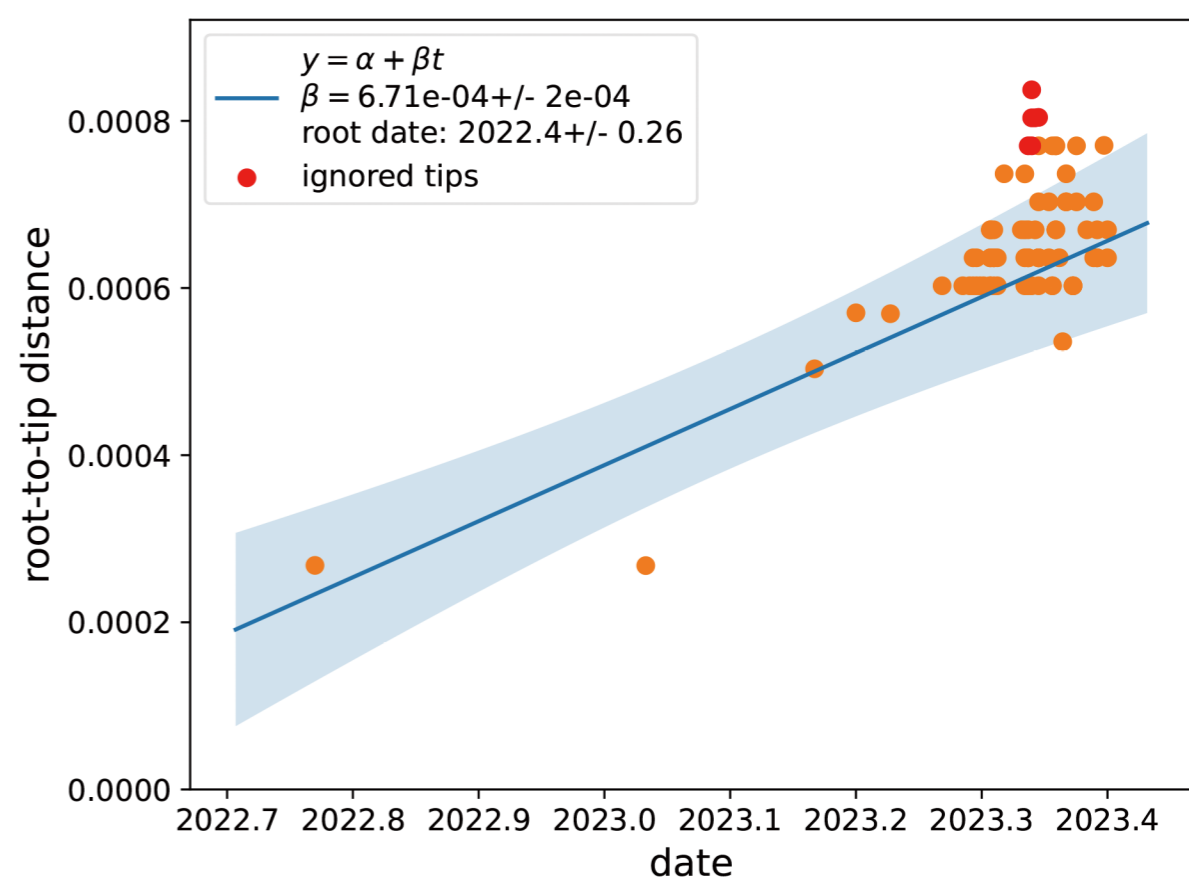**C****XBB.1.9.1\***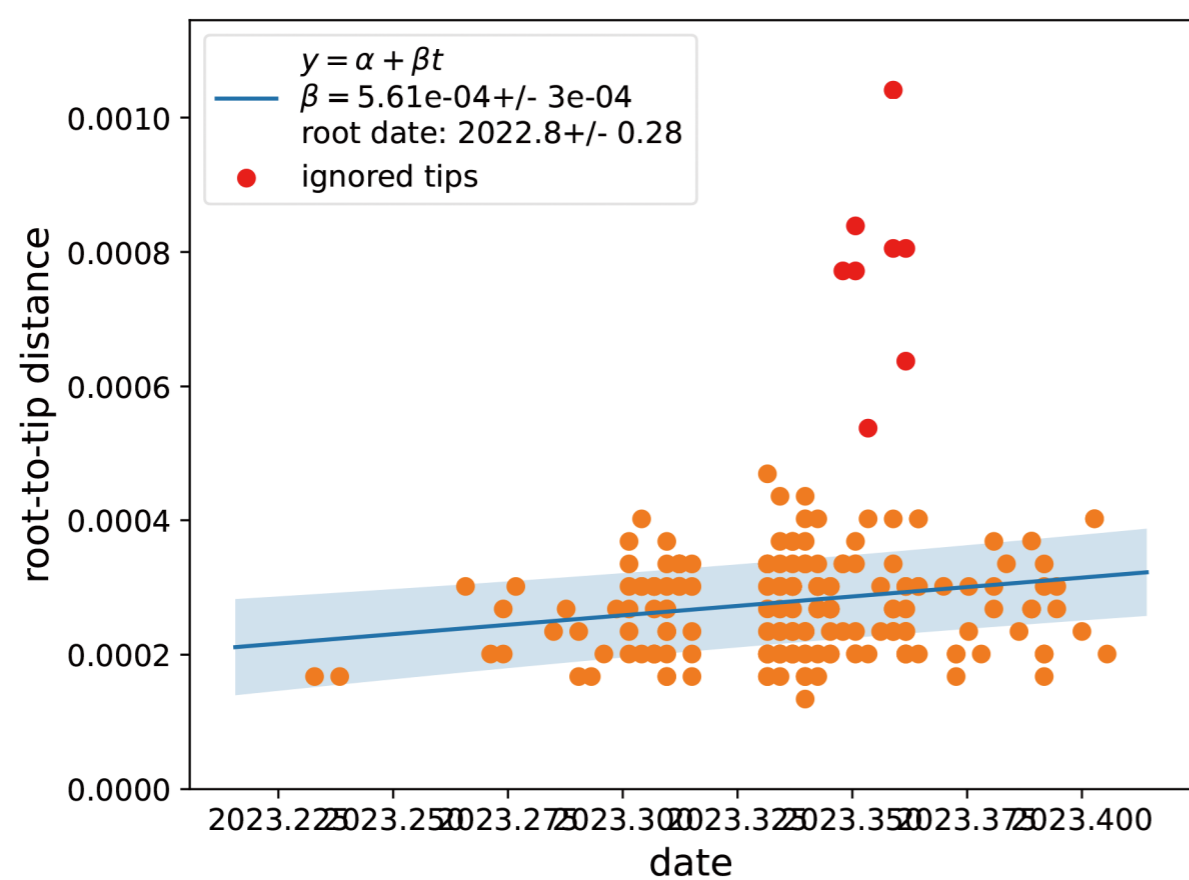**D****XBB.1.5\***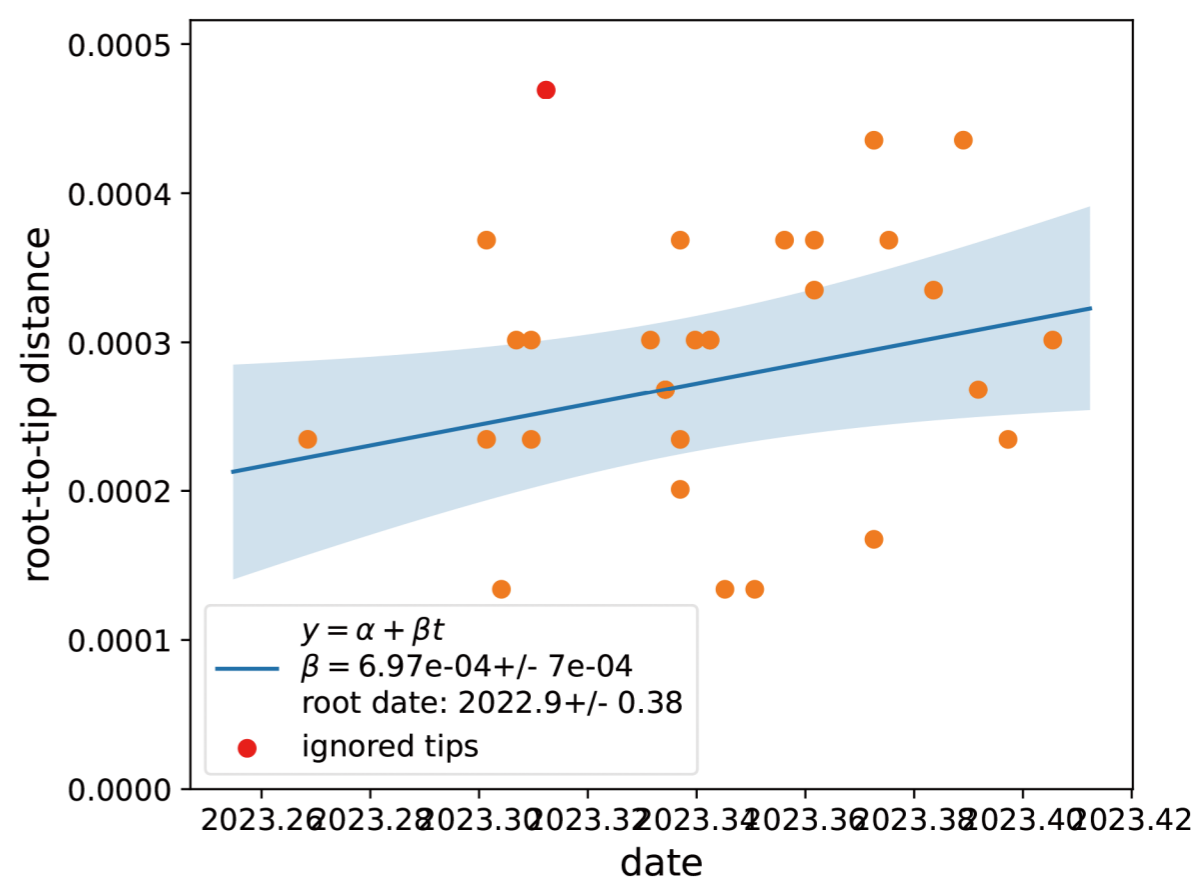**E****XBB.1.16\***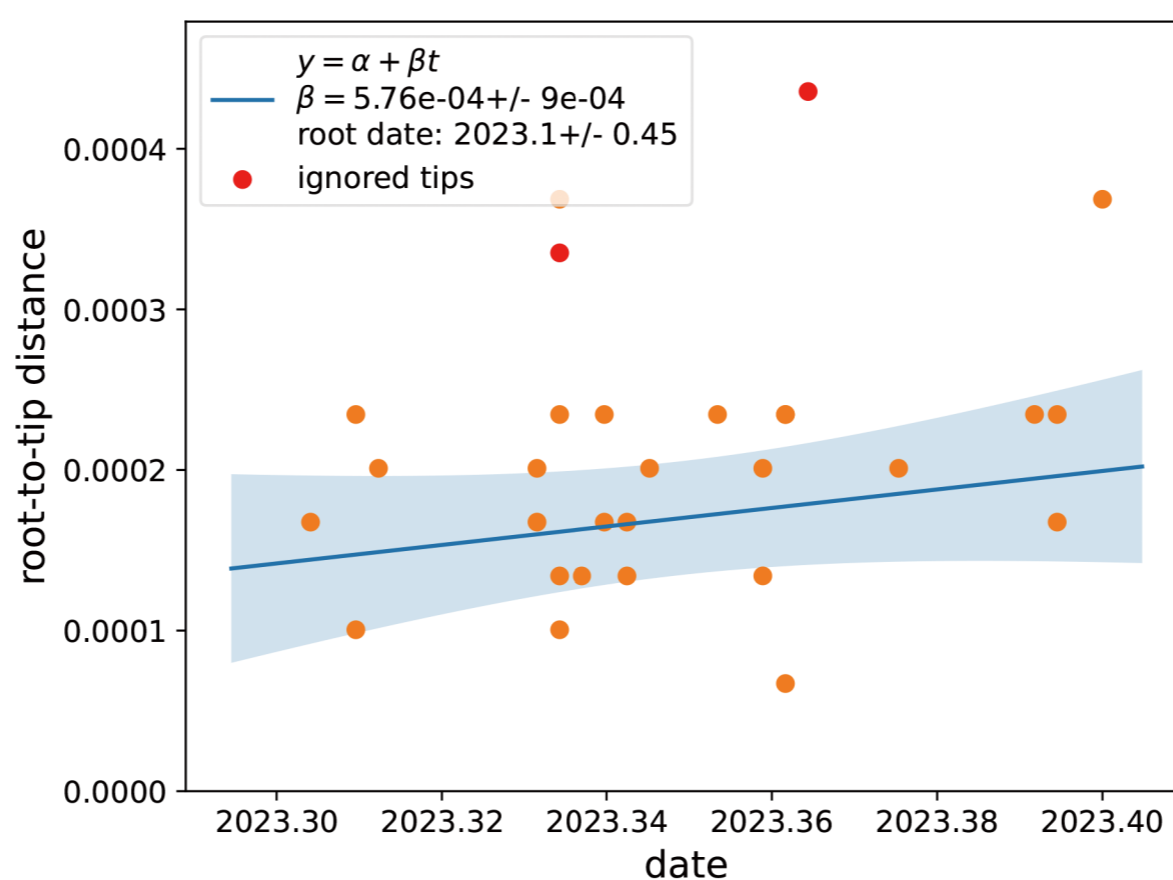

Supplement: Supplementary file 1 — Additional file 1. Fig. S1. Root-to-tip regression analyses on the maximum likelihood (ML) trees generated for original dateset comprising BA.5.2.48 (A), BA.2.75 (B), XBB.1.9.1 (C), XBB.1.5 (D), and XBB.1.16 (E) lineages. [file 12985_2023_2279_MOESM1_ESM.pdf]
