# Supplementary material for: A regional genomic surveillance program is implemented to monitor the occurrence and emergence of SARS-CoV-2 variants in Yubei District, China
Source: Virol J. 2024 Jan 8;21:13. doi: 10.1186/s12985-023-02279-6 (PMC10775548; doi:10.1186/s12985-023-02279-6)
Supplement: Supplementary file 2 — Additional file 2. Fig. S2. Phylogenetic tree in the time-scaled of 578 SARS-CoV-2 genomes sampled sequenced and 202 SARS-CoV-2 genomes sampled download from GISAID between January 2022 and May 2023 classification by divisions. GISAID, Global Initiative on Sharing All Influenza Data. [file 12985_2023_2279_MOESM2_ESM.pdf]

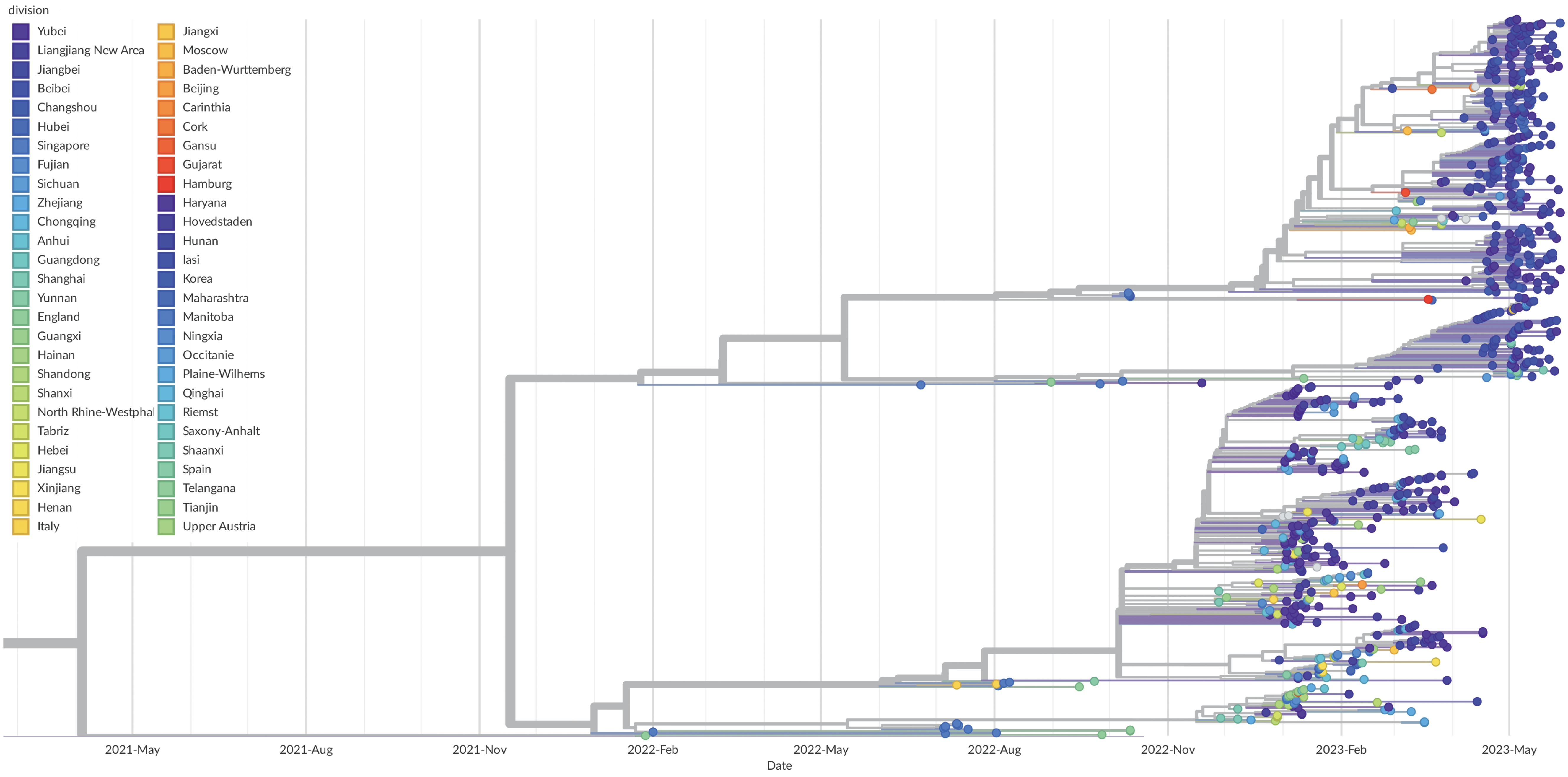
division
Yubei
Liangjiang New Area
Jiangbei
Beibei
Changshou
Hubei
Singapore
Fujian
Sichuan
Zhejiang
Chongqing
Anhui
Guangdong
Shanghai
Yunnan
England
Guangxi
Hainan
Shandong
Shanxi
North Rhine-Westphal
Tabriz
Hebei
Jiangsu
Xinjiang
Henan
Italy
Jiangxi
Moscow
Baden-Wurttemberg
Beijing
Carinthia
Cork
Gansu
Gujarat
Hamburg
Haryana
Hovedstaden
Hunan
Iasi
Korea
Maharashtra
Manitoba
Ningxia
Occitanie
Plaine-Wilhems
Qinghai
Riemst
Saxony-Anhalt
Shaanxi
Spain
Telangana
Tianjin
Upper Austria
2021-May
2021-Aug
2021-Nov
2022-Feb
2022-May
2022-Aug
2022-Nov
2023-Feb
2023-May
Date
